# An Artificial Intelligence Approach to the Craniofacial Recapitulation of Crisponi/Cold-Induced Sweating Syndrome 1 (CISS1/CISS) from Newborns to Adolescent Patients

**DOI:** 10.3390/diagnostics15050521

**Published:** 2025-02-21

**Authors:** Giulia Pascolini, Dario Didona, Luigi Tarani

**Affiliations:** 1 Genetic Counselling Unit, Istituto Dermopatico Dell’immacolata, IDI-IRCCS, Via dei Monti di Creta 104, 00167 Rome, Italy; 2 Rare Diseases Center, Istituto Dermopatico Dell’immacolata, IDI-IRCCS, 00167 Rome, Italy; d.didona@idi.it; 3 Department of Maternal Infantile and Urological Sciences, Sapienza University, 00161 Rome, Italy; luigi.tarani@uniroma1.it

**Keywords:** Artificial Intelligence (AI), Crisponi/cold-induced sweating syndrome 1 (CISS1/CISS; MIM#272430), Face2Gene (F2G), *CRFL1*, thermoregulation dysfunction, anhidrosis, sweat glands, ectodermal dysplasia-like phenotype

## Abstract

**Background/Objectives**: Crisponi/cold-induced sweating syndrome 1 (CISS1/CISS, MIM#272430) is a genetic disorder due to biallelic variants in *CRFL1* (MIM*604237). The related phenotype is mainly characterized by abnormal thermoregulation and sweating, facial muscle contractions in response to tactile and crying-inducing stimuli at an early age, skeletal anomalies (camptodactyly of the hands, scoliosis), and craniofacial dysmorphisms, comprising full cheeks, micrognathia, high and narrow palate, low-set ears, and a depressed nasal bridge. The condition is associated with high lethality during the neonatal period and can benefit from timely symptomatic therapy. **Methods**: We collected frontal images of all patients with CISS1/CISS published to date, which were analyzed with Face2Gene (F2G), a machine-learning technology for the facial diagnosis of syndromic phenotypes. In total, 75 portraits were subdivided into three cohorts, based on age (Cohort 1 and 2) and the presence of the typical facial trismus (Cohort 3). These portraits were uploaded to F2G to test their suitability for facial analysis and to verify the capacity of the AI tool to correctly recognize the syndrome based on the facial features only. The photos which passed this phase (62 images) were fed to three different AI algorithms—DeepGestalt, Facial D-Score, and GestaltMatcher. **Results**: The DeepGestalt algorithm results, including the correct diagnosis using a frontal portrait, suggested a similar facial phenotype in the first two cohorts. Cohort 3 seemed to be highly differentiable. The results were expressed in terms of the area under the curve (AUC) of the receiver operating characteristic (ROC) curve and *p* Value. The Facial D-Score values indicated the presence of a consistent degree of dysmorphic signs in the three cohorts, which was also confirmed by the GestaltMatcher algorithm. Interestingly, the latter allowed us to identify overlapping genetic disorders. **Conclusions**: This is the first AI-powered image analysis in defining the craniofacial contour of CISS1/CISS and in determining the feasibility of training the tool used in its clinical recognition. The obtained results showed that the use of F2G can reveal valid support in the diagnostic process of CISS1/CISS, especially in more severe phenotypes, manifesting with facial contractions and potentially lethal consequences.

## 1. Introduction

Crisponi/cold-induced sweating syndrome 1 (CISS1/CISS; MIM#272430) is a rare autosomal recessive disorder caused by biallelic variants in the cytokine receptor-like factor 1 gene (CRFL1, MIM*604237) [1], located on chromosome 19p13 and encoding cytokine receptor-like factor-1 (CRLF1), a member of the ciliary neurotrophic factor receptor pathway [2].

CRLF1 forms a complex with the cardiotrophin-like cytokine factor 1 (CLCF1, MIM*607672), which is secreted and acts on cells expressing the ciliary neurotrophic factor receptor (CNTFR). The CRLF1/CLCF1/CNTFR binding leads to the activation of the JAK/STAT signaling pathway, which supports the differentiation and survival of several neuronal cell types during development and adulthood. Interestingly, this complex mediates the switch of the noradrenergic/cholinergic phenotype of the sympathetic innervation of the sweat glands during postnatal development [3].

**Figure 1:**
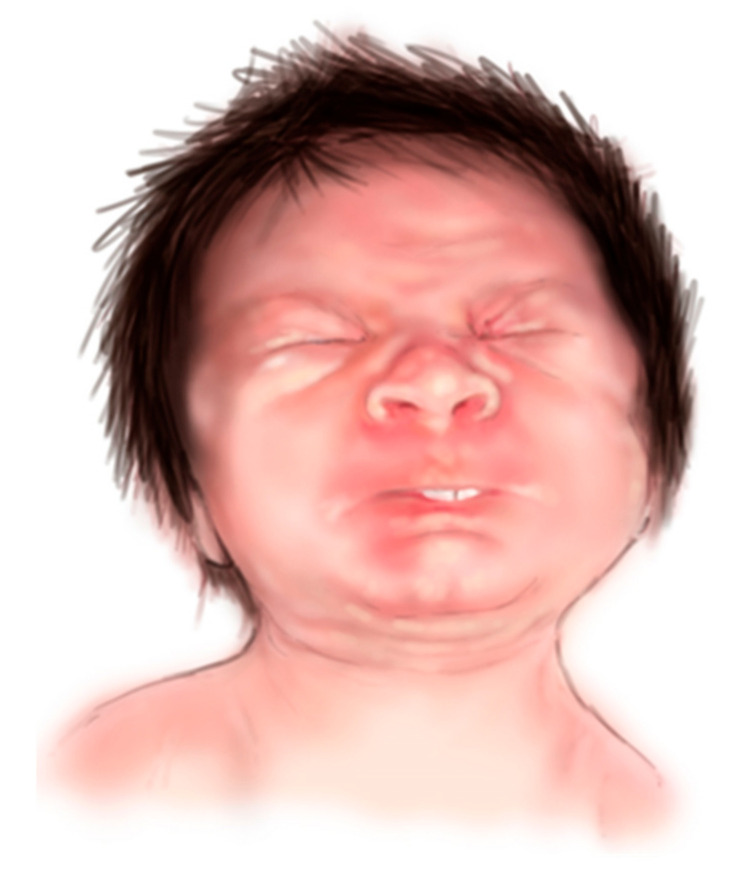
Artistic reproduction of the typical facial muscle paroxysmal contraction in response to handling or crying. The portrait has been drawn by the illustrator Susanna Brusa.

CISS1 cardinal features are recognizable at different ages and represented by neonatal orofacial weakness and impaired sucking and swallowing, causing feeding and respiratory difficulties [4]. In this period, facial muscle contractions accompanied by trismus, abundant salivation, and opisthotonus typically occur in response to tactile stimuli or during crying (Figure 1). In addition, affected infants can manifest spiking fevers in the first year of life. All these characteristics can result in early death in the absence of care [4]. The severe phenotype tends to abate after the first 24 months of age, although impaired thermoregulation and abnormal sweating (cold-induced hyperhidrosis) can persist. The clinical picture can evolve over time in a distinctive malformation pattern, mainly including skeletal anomalies (camptodactyly, progressive kyphoscoliosis) and craniofacial dysmorphisms [4]. These mainly comprise a round face, full cheeks, micrognathia, high and narrow palate, low-set ear, and depressed nasal bridge, representing a diagnostic handle [1,4]. Skin features, including ectodermal dysplasia-like phenotype and cracked hand/feet, have been reported [5].

In this research, we performed the first study of AI-powered image analysis in defining the craniofacial contour of the CISS1/CISS-associated craniofacial phenotype and of the feasibility of training the used tool in its clinical identification. We used Face2Gene (F2G, FDNA Inc., Boston, MA, USA; www.face2gene.com, accessed on 30 November 2024).

## 2. Materials and Methods

### 2.1. Artificial Intelligence (AI) Analysis

Three different algorithms present in F2G were utilized on previously published CISS1/CISS patients.

#### 2.1.1. DeepGestalt Algorithm

This algorithm is a facial image analysis framework, quantifying similarities to hundreds of syndromes. It is trained on a dataset of over 17,000 images representing more than 200 syndromes through a community-driven phenotyping platform and is based on machine-learning deep convolutional networks (DCNNs) for syndrome classification, as previously demonstrated [6].

The patient’s clinical portraits were selected from the medical literature by identifying scientific manuscripts indexed on the online free resource database Pubmed (https://pubmed.ncbi.nlm.nih.gov/about/, accessed on 30 November 2024) [5,7,8,9,10,11,12,13,14,15,16,17,18,19,20,21,22]. The keywords considered were Crisponi syndrome type 1, CISS1/CISS, cold-induced sweating syndrome 1, and *CRFL1*. Three independent researchers performed this phase of this study. Images of adult patients were not included because of their paucity, which did not permit the analysis in F2G, which requires a minimum of 15 photos. The 75 frontal images we identified were initially uploaded to F2G CLINIC to verify their suitability for the analysis with DeepGestalt technology (version DG 26.1.0.). The system works well in examining frontal portraits at a good resolution and possibly without additional objects (glasses, for example, or other furnishings). A total of 62 images were analyzable. We created three groups of facial portraits based on age (Cohort 1, 0–5 years, 19 patients, and Cohort 2, 6–15 years, 13 patients) and the presence of the typical facial trismus (Cohort 3, 30 patients). The list of patients for the three cohorts is available in Table 1.

Then, to compare the three cohorts, we interrogated the F2G RESEARCH using the same DeepGestalt technology but in a controlled environment, in which the algorithm is trained with half of each group and evaluated on the other half.

**Figure 2:**
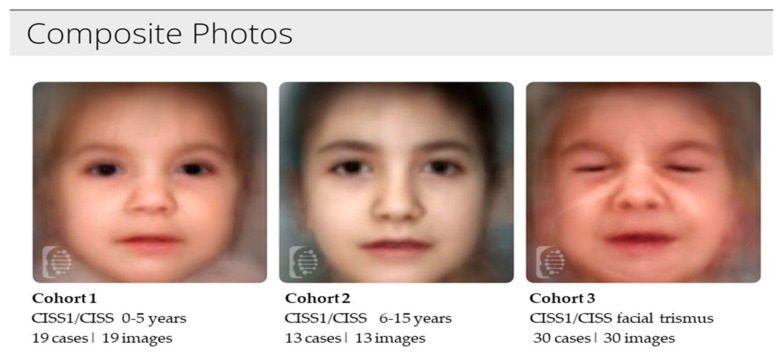
**DeepGestalt analysis of the studied cohorts and composite photos.** Composite photos of the three groups of patients generated by F2G. These have been obtained using F2G RESEARCH, after the upload of a frontal image into the CLINIC section, which automatically identified the suitable portraits for the experiment.

**Figure 3:**
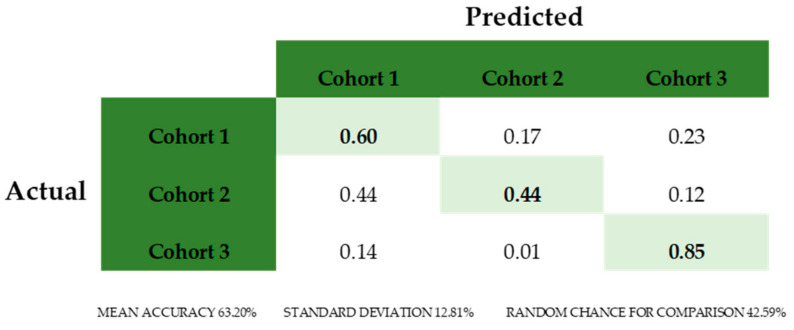
Multiclass Comparison and Confusion Matrix. In the Confusion Matrix, the True Positive (IP) values are highlighted diagonally while errors (false positives and false negatives) are reported in other rates. TP values of Cohorts 2 and 3 are significantly higher than the random chance for comparison and Cohort 2 is lower, indicating no recognition by the algorithm.

**Figure 4:**
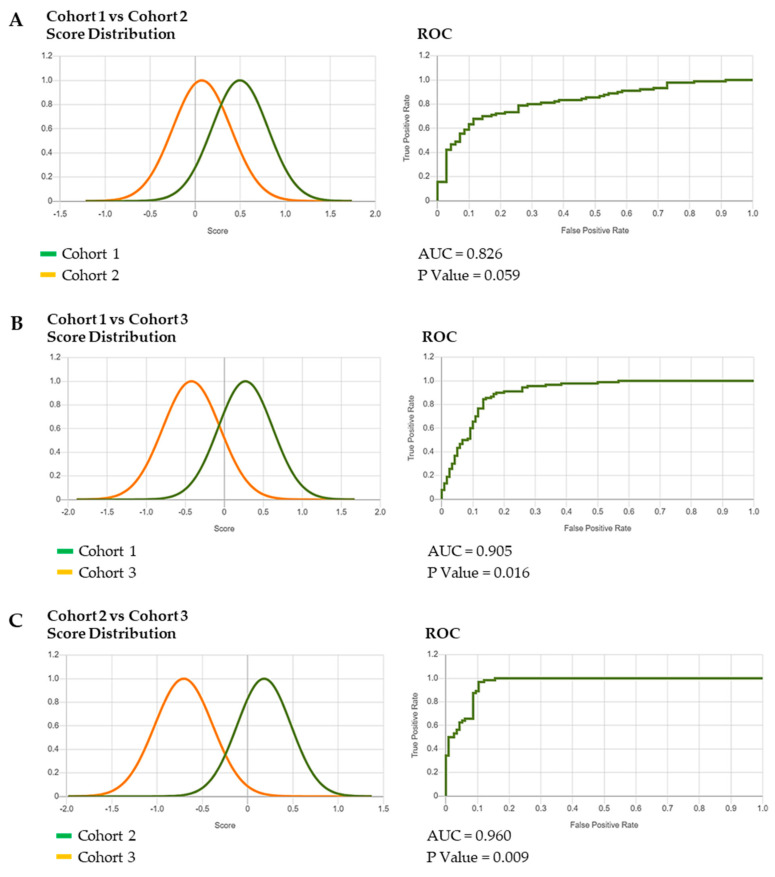
(**A**–**C**) **Binary comparison experiments of the three cohorts**. The results of the binary comparison between Cohort 1 and 2 (**A**), Cohort 1 and 3 (**B**), Cohort 2 and 3 (**C**), including the AUC and *p* Value are shown.

**Figure 5:**
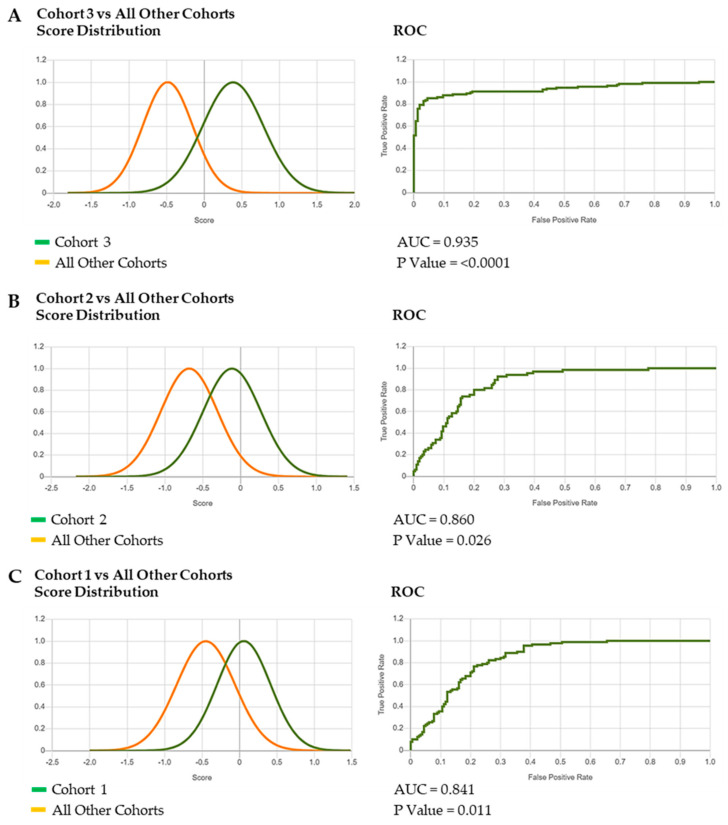
(**A**–**C**) **Binary comparison experiments of the three cohorts between themselves.** A greater separation of the two curves is observable in the comparison of Cohort 3 with the other two (**A**). Conversely, the comparison of Cohorts 1 and 2 with the other is characterized by overlapping curves (**B**,**C**).

This generated composite photos of the three studied cohorts (Figure 2), a Multiclass Comparison depicted as a Confusion Matrix, showing the True Positive (TP) values and errors (false positives and false negatives) (Figure 3) and ROC curves that allow for the calculation of the area under the curve (AUC) and corresponding *p* Values (Figure 4A–C and Figure 5A–C).

**Figure 6:**
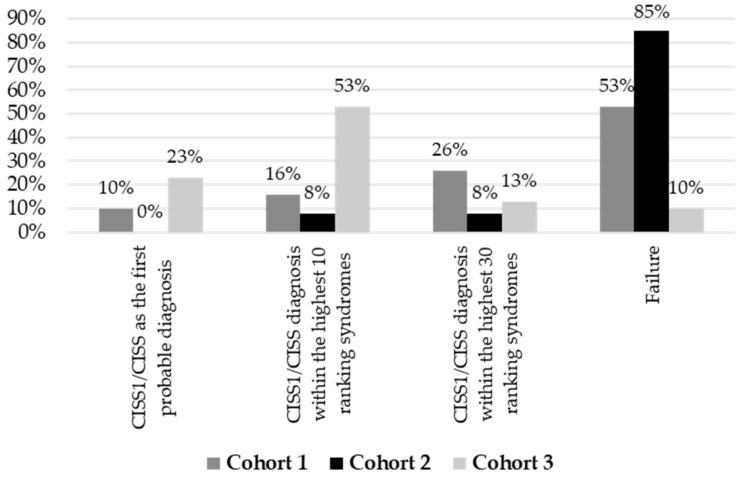
**DeepGestalt performance on CISS1/CISS facial recognition**. By uploading one frontal image in F2G CLINIC, the capacity of the platform to recognize the correct diagnosis has been tested for the three cohorts. The results are displayed as a failure, CISS/CISS recognition within the highest 10 and 30 ranking syndromes, and as the first probable diagnosis.

The CLINIC application was also used to verify the capacity of the system to identify CISS1/CISS as the first probable diagnosis and among the 10 and 30 highest-ranking syndromes, only examining the facial findings (Figure 6).

#### 2.1.2. Facial D-Score Algorithm

To further expand the AI study, we also utilized the novel Facial D-Score algorithm, which has been trained to differentiate between 2 classes of children’s frontal facial photos: images of patients diagnosed with a rare genetic disease and presenting facial dysmorphia, and an equivalently sampled second class of images of unaffected individuals [23]. This algorithm has been trained to work only on pediatric patients.

In the DS 2.6.0 version used for this analysis, the relevant threshold is 0.75, the value above which the portrait is predictive of an individual presenting facial dysmorphia.

#### 2.1.3. GestaltMatcher Algorithm

In addition, we utilized a third algorithm, GestaltMatcher version GM 2.6.0, to further analyze our cohort of cases.

The GestaltMatcher algorithm is a computational tool that converts facial characteristics captured by a photograph into numerical descriptors, allowing for quantitative comparison and clustering of facial phenotypes. The tool is part of F2G, a HIPAA-compliant platform that enables the interaction of different algorithms to analyze patients’ facial phenotypes and can be leveraged for syndrome classification (lump and splitting), phenotype delineation, and patient clustering based on facial phenotype [24,25,26].

**Figure 7:**
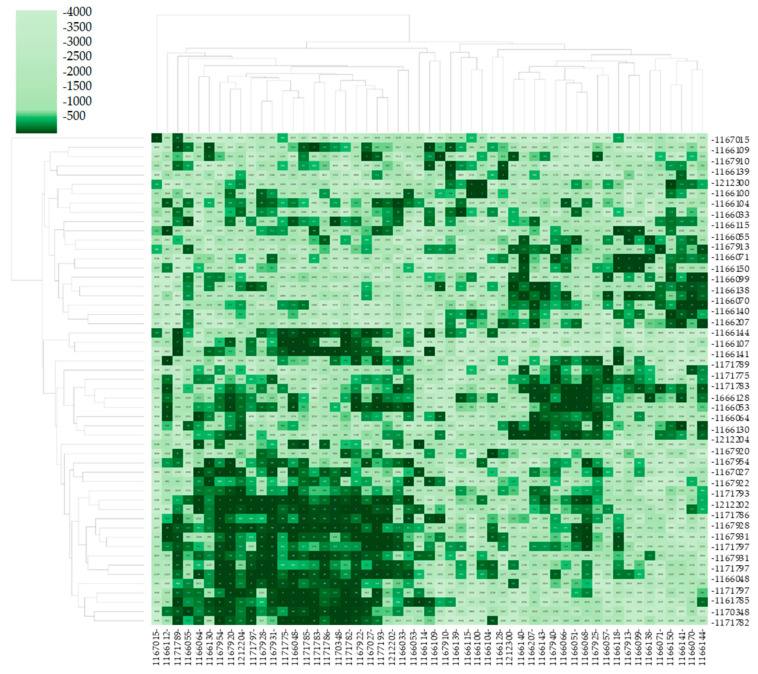
**GestaltMatcher experiment results and Pairwise Comparison Matrix (PMC).** Our cohorts’ images were compared with FDNA’s GestaltMatcher gallery (4300 images), outputting similarity ranks. Furthermore, a clustering method has been applied to the matrix (represented by a dendogram), clustering similar ranks together. The number in each of the matrix cells represents the similarity rank achieved. Dark green values (low rank) indicate higher similarity in facial phenotypic features within the test cohort.

In the Pairwise Comparison Matrix (PCM), our previously described cohorts’ images were compared with FDNA’s GestaltMatcher gallery (4300 images), outputting similarity ranks. Furthermore, a clustering method has been applied to the matrix (represented by a dendrogram), clustering similar ranks together (Figure 7).

**Figure 8:**
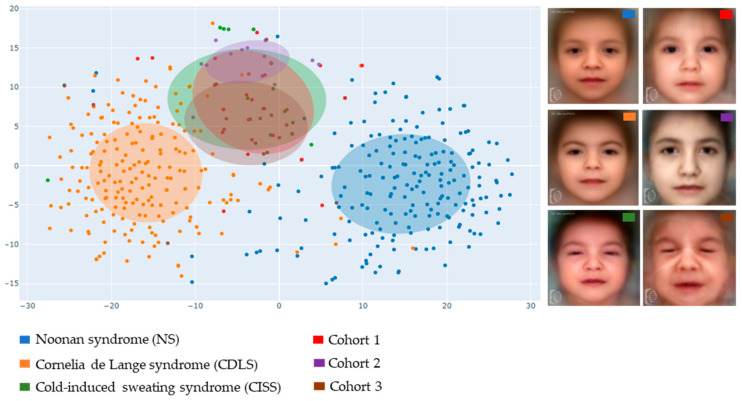
**GestaltMatcher analysis and t-SNE visualization**. The 3 most similar syndromes (NS in blue, CDLS in orange and CISS in green) to our 3 cohorts (Cohort 1 in red, Cohort 2 in violet, and Cohort 3 in brown) when compared to the GestaltMatcher database, are shown.

In the t-SNE visualization, by using a dimensionality reduction method to condense the numerical descriptors to just 2 dimensions, we obtained a 2D scatterplot. Each data point corresponds to a patient photo. This visualization includes the target cohort and the top 3 similar rare syndromes, each with at least 7 patient photos available in the GestaltMatcher database (Figure 8). The confidence ellipses had a radius of one standard deviation from each cluster’s centroid. The closer these data points are, the more similar their facial dysmorphology is.

## 3. Results

### 3.1. DeepGestalt Algorithm

The binary comparison between the three groups of patients showed an evident overlap of Cohort 1 and Cohort 2 (Figure 4A), while a partial superimposition of the two curves is recognizable for Cohort 3 compared with the other two (Figure 4B,C). All these observations are supported by the AUC and *p*-Value results (Figure 4A–C).

Comparing all the cohorts among themselves, we can observe that the algorithm better discriminates between Cohort 3 and all others (Figure 5A). A relative differentiation can be appreciated for Cohort 1 from the other two (Figure 5B), while Cohort 2 is poorly distinct (Figure 5C).

We also tested the algorithm for the facial recognition of CISS1/CISS in the three groups of patients using F2G CLINIC. The system did not identify the correct diagnosis mostly in Cohorts 2 and 1, while in Cohort 3, the failure was less consistent (Figure 6). The recognition within the 30 highest-ranking syndromes was achieved principally in Cohorts 1 and 2; the disorder was diagnosed within the 10 highest-ranking syndromes only in Cohorts 1 and 2 (Figure 6). The system diagnosed CISS1/CISS as the first most probable, especially in Cohort 3, and failed in Cohort 2 (Figure 6).

### 3.2. Facial D-Score Algorithm

The results of the Facial D-Score analysis were available for 57 photos with a score > 0.75 for 55 patients. Two subjects had the respective scores of 0.66 and 0.1.

### 3.3. GestaltMatcher Algorithm

For the GestaltMatcher experiment, a PCM with a clustering dendrogram was computed from 52 out of our 62 patients (Figure 7).

In the PCM, dark green values (lower ranks) indicate higher similarity in facial phenotypic features within the test cohorts. For example, we can see that if in the X and Y axes, the case IDs are the same, the value is 0, representing the similarity rank achieved when each photo is compared to itself (Figure 7).

Overall, we can see dark green values for all patients. Even when splitting the cohort via the top clustering level in the dendrogram, the small cluster of one patient to the right still achieves high similarity with the rest of our cohort. These observations indicate that the cohort forms a well-defined cluster of facial dysmorphology similarities (Figure 7).

In the t-SNE representation, we visualize the three most similar syndromes to our cohort when compared to the GestaltMatcher database. These conditions are Noonan, (NS), Cornelia de Lange (CDLS), and cold-induced sweating syndromes (CISS). Interestingly, our cohort appears to form a distinctive cluster, mostly separate from the other most similar syndromes, with a slight overlap with CISS (Figure 8), as expected.

## 4. Discussion

In recent years, AI tools have been introduced into the medical community to support and facilitate diagnostic processes. These have been successfully applied in the field of rare diseases, especially in the diagnosis of genetic syndromes with craniofacial anomalies. Often, these present as complex and ultrarare disorders or with subtle or atypical dysmorphic notes, which can complicate their identification.

On the other hand, several genetic conditions are characterized by pathognomonic clinical signs, and the AI application can confirm a clinical suspicion, supporting the molecular diagnostic process.

Recently, a novel machine-learning technology, namely Face2Gene (F2G), has been made available online for clinicians, and it deals with malformation disorders, mostly at pediatric ages. Indeed, a pediatrician view has been recently introduced, including a newly developed technology, Facial D-Score, that supports the decision to refer the patient to a genetic diagnostic workup, providing a guideline about the presence of facial dysmorphisms.

F2G contains some algorithms, such as DeepGestalt technology, the first developed and most used, which is trained on thousands of images of patients with genetic disorders, quantifying similarities to hundreds of syndromes [6]. It can differentiate unaffected from affected individuals or distinguish one specific syndrome from several others, with binary experiments [6].

Over time, many studies have experimented with this technology in a clinical setting. In this regard, the craniofacial phenotype of genetically heterogeneous syndromes such as chromatin disorders [26,27], as well as rare genetic diseases with skin and annex involvement in a specific age [28,29], has been investigated.

With this approach, a specific syndromic condition has been studied in diverse populations [30] or in single ethnic groups [31] or medical centers [32].

In the present research, we have applied for the first time this AI-driven facial phenotyping to better define the face of CISS1/CISS. This is a rare genetic syndrome with potentially lethal consequences in newborns, mostly due to severe thermoregulation dysfunctions [4] and caused by deleterious variants in the *CRFL1* gene [1]. The latter is implicated, through binding with its ligand CLCF1, in several biological processes through JAK/STATs, MAPK, and PI3K signaling pathway activation, controlling cell growth, differentiation, maturation, and apoptosis during embryo development [3].

If we look at the main typical clinical features of CISS1, or else facial muscle contractions and thermoregulation anomalies, previous studies have demonstrated a correlation with the molecular base of the condition. Indeed, the impaired orofacial muscle contraction and orofacial weakness can be explained by the *CRLF1/CLCF1* impairment, the loss of function of which is related to the paucity of motor neurons in the Central Nervous System (CNS) [3]; additionally, *CRLF1* perturbations can be associated with abnormal relaxation of the lower esophageal sphincter [3].

On the other hand, although the mechanisms underlying the severe thermal rise in neonates often causing premature death have not been completely unraveled, the alterations of the CRLF1/CLCF1/CNTF complex in mediating the switch from noradrenergic to a cholinergic differentiation of sweat gland innervation can be considered the main causative determinant in the more tardive cold-induced sweating [1,4]. Interestingly, the impaired thermoregulation could suggest a clinical overlap with the ectodermal dysplasia clinical spectrum. Thus, if CISS1/CISS is a genetic condition with a recognizable skin phenotype may represent an emerging field of research, requiring further investigations.

A molecular relationship is identifiable also in the CISS1 skeletal phenotype resulting in kyphoscoliosis, hand camptodactyly, cubitus valgus, joint contractures, and facial dysmorphisms, highlighting the involvement of *crfl1* and *clcf1* in mouse bone development [3].

Taken together, these observations point out again the phenotypic complexity of CISS1/CISS, which is then a multiple congenital anomaly syndrome with systemic features and craniofacial involvement, configuring peculiar faces. This has been strongly supported by our Facial D-Score values.

The results we have obtained from the DeepGestalt analysis indicate an acceptable performance of the tool in the recognition of the typical facial muscle contractions triggered by tactile and crying-inducing stimuli. Indeed, the findings of the binary comparison experiments are suggestive of a similar craniofacial appearance of CISS1/CISS at different ages (Cohorts 1 and 2) (Figure 4A,B), while the “facial grimace” (Cohort 3) was clearly distinguishable, as demonstrated by the separation of the two curves (Figure 4C). This was also verified in the binary experiments comparing the three cohorts: Cohort 3 remains the most recognizable group of patients (Figure 5A–C).

The use of F2G CLINIC in the correct identification of CISS1/CISS by only analyzing frontal portraits is also in good agreement with these results. Indeed, we registered the capacity to recognize the disorder as the first most probable diagnosis, especially in Cohort 3 (Figure 6). Regarding the other two groups of patients, Cohort 1 seems to be more identifiable than Cohort 2 (Figure 6). This could be associated with a more distinctive facial phenotype, suggesting mitigation of the facial dysmorphisms proceeding with time. The failure we have observed can be in part related to the presence in the analyzed images of medical devices such as tubes, interfering with a correct facial analysis. Clearly, the algorithm needs further training to identify this condition with higher accuracy, to which this study largely contributed. Results from additional AI tools, such as Natural Language Processing (NLP) algorithms analyzing clinical notes could aid these kinds of tools in achieving a higher degree of identification.

The Facial D-Score results suggested the presence of facial dysmorphology for most patients.

The findings of the GestaltMatcher experiments pointed to homogenous dysmorphic traits in the studied cohort (Figure 7). Concerning the three similar genetic disorders, the identification of CDLS could suggest shared dysmorphic characteristics: coarse facial traits can link CDLS and CISS1. Conceivably, the presence of apparent hypertelorism in some patients may result in NS identification. The strong overlap with CISS further supports the concept that CISS1 and CISS could represent a single clinical entity, as previously postulated [12].

## 5. Conclusions

CISS1/CISS is a rare multisystemic condition that can present difficulties in its recognition and diagnosis. It is known that the neonatal period is the most complicated and dangerous for these patients, being associated with potentially lethal consequences due to thermoregulatory issues. Therapeutic devices can be used to control severe symptoms, although specific drugs have not yet been developed. Thus, physicians who are not familiar with such uncommon phenotypes or who do not frequently deal with rare diseases may need the support of an automated facial recognition instrument in the early detection of the disorder, expediting the diagnosis as well as the initiation of a timely lifesaving therapy. In the present research, computer vision analysis proved to be useful in the correct identification of the typical facial trismus associated with the most severe expression of the syndrome.

Moreover, this study contributed to training F2G in recognizing CISS1/CISS, newly taking part in the model, and represents the first application of a machine-learning technology in this syndrome. Certainly, the technology must be improved for the analysis of this disorder, as demonstrated by the non-optimal *p* values. Thus, a possible future goal will be its improvement and possibly the inclusion of other AI tools, such as NLP for clinical notes and the automated extraction of HPO terms to combine with image analysis.

The AI-powered image analysis can support clinicians in identifying CISS1/CISS phenotypes and may be applied in the future to large and age-diversified patient cohorts, including newborns.

## Figures and Tables

**Table 1:** A list of the patients in the three studied cohorts. The single case reports are reported as “Patient 1”. The references are between brackets. Some patients were selected at different ages as indicated by an asterisk.

CISS1/CISS		
Cohort 1 0–5 Years	Cohort 2 6–5 Years	Cohort 3 Facial Muscle Contraction
Patient 1 [7]	Patient CS07 [1]	Patient C4 [4]
Patient 1 [8]	Patient CS10 [1]	Patient H13 [4]
Case 3 [10] *	Patient CS14 [1]	Patient H14 [4]
Case 4 [10] *	Case 3 [10] *	Patient I14 [4]
Patient 1 [11] *	Case 4 [10] *	Patient J15 [4]
Patient 5 [12] *	Patient 1 [11] *	Patient K16 [4]
Patient 1 [13]	Patient 2 [15]	Patient CS03 [1]
Patient 1 [15] *	Patient 3 [17]	Patient CS37 [1]
Patient 1 [15] *	Patient 1 [21]	Patient 1 [8]
Patient 2 [15]	Case 2 [22]	Patient 1 [9]
Patient A [18] *	Case 3 [22] *	Case 4 [10] *
Patient A [18] *	Case 4 [22]	Patient 5 [12] *
Patient B [18] *	Case 5 [22]	Patient 1 [14]
Patient C [18] *		Patient 1 [16]
Patient C [18] *		Family 1 patient V1 [5]
Patient 1 [19] *		Family 2 patient V2 [5]
Patient 3 [20]		Family 3 patient V6 [5]
Case 1 [22]		Family 4 patient IV1 [5]
Case 7 [22] *		Patient 1 [17]
		Patient 2 [17]
		Patient A neonatal [18] *
		Patient A infancy [18] * Patient B [18] *
		Patient C [18] *
		Patient 1 [19] *
		Patient 1 [20]
		Case 3 [22] *
		Case 7 [22] *
		Case 13 [22]
		Case 14 [22]

## Data Availability

The data in this study are available from the corresponding author upon reasonable request due to privacy restrictions.
